# Neocolonialism and science diplomacy: personal reflections from the Middle East on mental health policy and practice

**DOI:** 10.3389/fpubh.2024.1409341

**Published:** 2024-09-19

**Authors:** Nadia Taysir Dabbagh

**Affiliations:** Mental Health Centre of Excellence, Al Jalila Children’s Hospital, Dubai, United Arab Emirates

**Keywords:** mental health, Middle East, neocolonialism, policy and practice, grassroots, narrative approach, patient first, clinical excellence

## Abstract

Neocolonialism has led to an imbalance in the production of knowledge and a clunky imposition of frameworks and models of practice that do not meet the needs of local communities. In contrast they can serve the central function of colonialism by draining valuable resources. Inequity of science diplomacy has diluted local voices and given precedence to colonialist knowledge and models of practice. It is argued that clinical, training and research excellence applies to those activities that meet and fulfil the clinical, training and research needs of the community in which they are embedded. Through personal reflection on contrasting Middle Eastern settings (Palestine and the UAE), the call is for the source of knowledge production and the driver for innovation to be daily clinical experiences listening to families in the community. This will result in policy and practices that are meaningful and impactful as illustrated by way of three examples: (1) a narrative approach to exploring suicide (2) an “all-hands-on-deck” clinical pathway for Autism assessments which transformed the lives of children and families with little additional resource, but with a fundamental shift in approach from “top down” to “bottom up” one as part of an organization-wide “Patient First” approach and (3) a rights-based, collective approach to developing mental health strategy. These examples are linked in terms of taking a shared “listening approach” but are applied to different levels moving from personal individual narratives to community clinical service to national strategy.

## Introduction

There is “no health without mental health” and, as the demand for mental health services, particularly for young people, surges in the 21^st^ century, mental health and well-being is high on government and policy-makers’ agendas (1). In 2018, Dubai endorsed its first Mental Health Strategy and in 2023 the State of Palestine endorsed its first National Strategy for Child and Adolescent Mental Health.^1^ It is argued here that clinical, training and research excellence applies to those activities that fulfil the clinical, training and research needs of the community in which they are embedded. Policies and practices borne out of local needs are more likely to succeed (2).

## Neocolonialism and science diplomacy

Neocolonialism is the use of economic, political, cultural, or other pressures to control or influence other countries. In mental health policy and practice this has led to the imposition of knowledge, frameworks and models of practice from the Global North that do not necessarily meet the needs of local communities. In contrast they can drain resources serving the central function of colonialism. Science diplomacy frameworks need to take the colonial heritage which undermines local knowledge into account so as to make the production of global knowledge more equitable. “We need to think about how we can collectively change the narrative so every emerging scientist from the Global South can flourish and have an equal opportunity to conduct research that is meaningful to them and their societies” (Dajani et al, Submitted Manuscript).

### Global citizenship

Global citizenship is the umbrella term for social, political, environmental and economic actions of globally minded individuals and communities on a worldwide scale (3).^2^ This concept was visibly called into question by the disproportionate impact of the COVID-19 pandemic and the failure to achieve either Sustainable Development Goals (SDGs) or basic human rights in the Global South. Dajani et al. again, “As global citizens we have failed to act on science diplomacy due to the implicit idea that to level the playing field, the ‘West’ must take on an old colonial pastoral role that undermines local knowledge and expertise.” Instead it props up a dominant “top down” approach (Dajani et al, Submitted Manuscript^3^). That said, the UAE was a shining exception in its effective combating of the COVID pandemic by quickly and nimbly adapting and implementing masks, hand hygiene, limiting group gatherings, rapid testing, vaccination campaigns and field hospitals.^4^

This paper reflects on neocolonialism and science diplomacy by way of three examples in two very different Middle Eastern settings - Palestine, an ancient land besieged by conflict and destruction where nowhere is safe, and Dubai (UAE), the land of optimism and construction and one of the safest places in the world. The first example pertains to gathering personal narratives at the individual level; the second example to developing clinical pathways at the service level, and the third to policy writing at the strategic national level. Illustrated through these examples, the argument is for us to gather *our* data, by our clinicians and researchers to build models that work for *our* communities.

### Example one: an “unframed” narrative approach to suicide research

The first example takes us to Palestine in the 1990’s. As a medical student conducting fieldwork for PhD research on the topic of suicide in Palestine, I was tasked with interviewing people following a deliberate act of self-harm (4). Having had no mental health training, I recall being paralysed by fear that I would do unintentional harm or face unexpected scenarios and be consumed by the sense of “not knowing.” Perhaps, especially for those who have chosen careers in a “caring profession,” the act of knowing and doing can be what gives us a sense of purpose and utility. We shy away from the unknown and the uncertain.

As it was, I sought guidance from an experienced clinical psychologist who gave me a few tips on how to conduct the interviews and we identified the key pieces of information to be gathered from all participants. Otherwise, I was left with just a few open-ended exploratory questions and a tape recorder.^5^ She offered psychological follow up to the research participants if requested. All I could do was listen with curiosity, care and empathy and thank the participants for their time. And what I did have at that stage of my life, was plenty of time. The recorded narratives were later poured over for hours to analyze the intonation, the choice of words and phrasing, and to elicit themes through a narrative approach on which my PhD thesis and later book were based.

At the time, what struck me about the experience beyond the narratives themselves, was my surprise when participants sincerely thanked me for listening to their story. I was humbled, having a guilty sense that I had gone in empty-handed. But perhaps the attentive ear and empathic heart had a value, and - again perhaps - I reflect now, they thanked me *because* it was uninterrupted without judgement or “correction.” Later in life, as a consultant child psychiatrist, I realised I had been initiated into the “gift of therapy;” sitting with uncertainty (with an attentive ear and an empathic heart) and allowing people to tell their stories, in their way, at their pace and their structure. In retrospect I also appreciated having had the gift of time, something that can be in short supply in a busy children’s hospital.

Psychiatrist and existential psychotherapist Irvin Yalom presents an egalitarian and nonhierarchical view of the therapist-client relationship, referring to both parties as “fellow travelers” in the “mysterious and challenging journey of life (5).” He talks of seeing each person as a unique constellation of all that they have experienced. In family therapy, the therapist is encouraged to lower or shift position whilst listening with “curiosity and circularity.” What is remarkable from such therapeutic stances is that - with patience and persistence - solutions unfold. An experienced, now retired, Eating Disorder consultant remarked that he always had one “impossible” patient whose needs and circumstances were beyond him and the team. They would confidently conclude that an insurmountable impasse had been reached and nothing more could be done to help. “But with time I realised that, a few months later, I would still have an ‘impossible’ patient but it was now a different one.” He recognised that the former impossible case had “shifted” into the realms of the possible.^6^

Thirty years, many professional exams, much training and thousands of patients later, when faced with complexity, putting frameworks aside and listening, I have faith this process can allow us ways forward, even if only with a single step. This single step can sometimes be all that is needed to shift the system and open up new horizons.

### Example two: an “all-hands-on-deck” approach to creating an autism pathway

The second example of taking a grassroots approach takes us to the UAE where, in 2023, I was invited to oversee a change management project for a specialist mental health service which was facing long waiting lists.^7^ Waiting times for a first appointment were running into years and there were daily complaints from distressed families who were unable to get their needs met. The previous team was diligently using imported Western models with intricate structured assessments and elaborate reports. This was deemed “best practice” in accordance with international benchmarking and standards. Any attempts at change were labelled at best “sub-standard” or “poor quality” and at worse “unethical” or “unprofessional.” Senior management was in a quandary as the department was well-resourced and yet the central task of meeting patients’ needs was not being achieved. This coincided with a time when patient satisfaction was high on the agenda, with an organization-wide campaign to put “Patient First.”

The first step was to meet with mothers and to listen carefully, taking notes of their concerns. The result was a long list of requests. After checking I had heard from everyone, I read back the list and asked, “What would you say is most important?” They were unanimous, “We want to be seen.” “And we want to be seen soon.” They did not mind if they could not see a highly specialized consultant or a full team of multidisciplinary professionals and they did not mind if the assessment report was not extensive, but *time* was important. “As a mother who has been told your child may have Autism, 1 day is too long to wait.” Her voice and message rang in my head for the months ahead.

The second step was to review complaints which were running at the highest for the mental health department in comparison to all other hospital departments. The most frequent complaint was the lack of access; that is, not being able to get an appointment to see a clinician. This mirrored the mothers’ group’s clear request to be seen face to face - and soon.

The importance of face-to-face exchange is common to most cultures and societies, but perhaps more so in Middle Eastern cultures, where meetings and greetings, and listening to concerns from the community has a long cultural tradition. As a clinician, one can find oneself taking on the role of a “host” welcoming families less so as “patients,” but rather as “guests” who are seeking refuge and advice during troubled times. This host-guest relationship is disrupted by too much formality, emails, letters and questionnaires and may be perceived as inhospitality in a culture which prides itself on hospitality and generosity.

The third step was to meet with the team. Several members were relieved to hear that there were changes afoot; they recognized there was a problem and that something had to be done differently. They enthusiastically embraced the changes with expertise and dedication. But another section of the team conveyed a different emotion: a palpable fear of seeing patients face-to-face and of straying from strict imported protocols and ways of working. In hindsight this was similar to the paralytic fear I had experienced as a novice medical student sent off to see suicide research participants: the fear of the uncertainty and the fear that harm could be done if “unarmed” with sufficient frameworks to lean on.

We soon realised that given the magnitude of the problem (at least 40% of referrals were for Autism assessments), we had to move away from one specialist multidisciplinary team dedicated to Autism which created bottlenecks and - even at full capacity - could only complete four Autism assessments a month (6). Rather, we made Autism “everyone’s business” with an “all-hands-on-deck approach.” Every mental health team member was expected to contribute. We divided into five clinical teams each with a consultant psychiatrist and licensed psychologist and/or social worker. We looked for willing partners outside the department and even the hospital. We found enthusiastic partners in the Primary Healthcare (PHC) sector; managers and clinicians who - being embedded in the community - were aware of the challenges facing families and were themselves frustrated by their referred patients not being seen in a timely manner. We also reached out to our neurodevelopmental paediatric colleagues who were also struggling to meet the demand for their services.

Reviewing international and local guidelines, we drew up a clinical pathway and then shared out the assessment task whereby the initial assessment was conducted face-to-face by five family physicians doing 1 day of assessments per week (7). We then shadowed and trained junior mental health team members to complete brief structured assessments using a proforma with a few checklists in the community PHC centres. More senior clinicians were designated as “diagnosticians” (child psychiatrists, neurodevelopmental paediatricians and specialist psychologists) who followed up after the initial assessment had been conducted, with an outreach clinic day in PHC, seeing four to six patients and issuing reports on the same day. By means of this model we were able to increase the numbers of completed assessments 10-fold (1000%) to approximately 40 per month and to reduce the waiting times from years to a matter of weeks or even days. Ten months later and there is no longer a waiting list for Autism assessments - although we are continuing to work on reducing the waiting time from referral to a completed assessment and increasing the offerings post-assessment with support groups and therapeutic interventions.

The development of a streamlined Autism pathway in the UAE transformed the lives of children and families with little additional resources, but with a fundamental shift in approach from “top down” to a grassroots “bottom up” and “Patient First” approach. At a meeting with hospital colleagues, one consultant asked, “Why should not they wait a few weeks? This is usual in the UK.” I reflected that “Yes, this is true in the models used and imported from the West, but the question is, why should they wait?” If their primary request is to be seen promptly, then the task for an organization committed to clinical excellence is to find a way to meet that need within the surrounding constraints. We made the day-to-day experience of working with families and listening carefully to their needs, into the ultimate source of knowledge production and a driver for innovation. It is argued that this resulted in strategies, policy and practice that were meaningful and impactful.

### Example three: a rights-based, collectivist approach to mental health strategy

The third example of taking a more collaborative, “listening” and bottom-up approach takes us back to Palestine and concerns the development of a mental health strategy. In 2022, in partnership with the UK-charity Medical Aid for Palestinians (MAP), the Royal College of Psychiatrists (RCPsych), UK,^8^ I was invited to develop a national strategy for child and adolescent mental health in Palestine for the Ministry of Health. The aim of the strategy was to set key priorities for child and adolescent mental health so that funders, institutions, organizations and community members could align their activities in a coordinated and efficient way.

It is known that children are at higher risk of developing mental illness when living in overcrowded areas with ongoing shelling, siege conditions and other acts of violence, as is the case in Gaza (8–10). A significant proportion of Palestinian children experience serious psychological distress, not wanting to be parted from their parents and suffering anxiety and stress-related conditions (11–13).

As a RCPsych volunteer, I was tasked to lead on the strategy development. Information was gathered from an extensive literature review and in-person missions to visit the West Bank to meet key stakeholders from governmental and non-governmental organizations including the Ministries of Health, Education and Social Development and the only Palestinian Child and Adolescent Mental Health Service in Hebron. A thematic meeting was held in August 2022 and a feedback meeting in December 2022. All comments and feedback were reviewed and incorporated into the final document for submission to the Ministry of Health for official approval.

As a testament to the Palestinian people’s ability to adapt, endure and demonstrate *sumud* (steadfastness), through strong family and community relations, many children showed remarkable resilience.^9^ However, already in 2022, Palestine’s children were bearing the burden of decades of violence, conflict and hardships that had accumulated during their lives and those of their parents and grandparents. In total, 2,242 Palestinian children had been killed by Israeli military forces between the years 2000 and 2022. Through the examination of Israeli policies towards Palestinian children, researchers concluded that childhood is not a given for Palestinian children, but instead something that must be determined, retrieved, and understood within a complex web of implications mandated by the dynamics of power that are in play (14). The central message of the strategy was thus an advocacy, “rights-based” approach. Entitled, “Every child deserves a childhood and every child deserves a future,” the national strategy took a holistic view of childhood and adolescence, using the multi-level framework for child and adolescent well-being developed by the United Nations Children’s Fund (UNICEF) (15).

It is a dark and painful irony that 2023, the year of publication of the first national strategy for child mental health, heralded the escalation of hostilities resulting in a genocidal onslaught on tens of thousands of children and families and the systematic destruction of homes, schools, playgrounds, mosques, churches and hospitals. This is causing untold childhood mental trauma that will reverberate for generations to come (16, 17). Since the strategy recognizes and addresses the underlying political, economic and social drivers and also identifies the policies to “unchild” children and take away their childhood, the strategy’s call for action “Every child deserves a childhood, every child deserves a future,” becomes even more relevant.

The vision is for every Palestinian child’s mental health and well-being to be promoted and protected throughout their developmental journey into adulthood by strong multi-sectorial support networks. Mental illnesses need to be detected and treated by collaborative, effective systems of care, free from stigmatization, discrimination and marginalization so the children can live fulfilling lives as integrated members of society. Collectivist approaches, including group therapeutic approaches, were embraced in recognition of the shared collective trauma that requires communal healing (18, 19). Built on the four pillars of Rights and Regulation, Prevention and Promotion; Capacity Building and Clinical Service, as well as Community Integration and Contribution, the vision can be realized through the implementation of 10 initiatives. Each of these has their own action plan and outcome measure, along with the critical enablers of funding and stakeholder participation and collaboration (20).

The strategy was developed at the invitation of and through the collaboration with key stakeholders, taking a rights-based approach to protect childhood and embracing collectivist approaches to reflect the needs of the population. It is an example of policy document development that comes from the community that it serves and yet at the same time speaks in a global language that can build bridges and attract funding for local projects.

### Looking ahead

Science diplomacy is an opportunity to change the narrative and gain access to knowledge, science and technology resources through building bridges with the relevant stakeholders in the Global North and South. The key objectives are:

1. Development of successful policies and clinical pathways with outcomes which are meaningful and impactful for the communities in which they are embedded.
2. Equality in the relationships between scientists, researchers and the communities they study, by taking up the stance of being a part of the community, working together to understand dilemmas and co-creating solutions to solve them.
3. Enhanced alignment and coordination between key sectors and stakeholders to increase equity in science diplomacy.
4. Ethical values that reflect *our* cultural, religious, political and social make up embedded in guidelines for conducting research and developing models of practice.
5. Innovative and “out of the box” thinking by maintaining a curious and listening approach in order to understand clinical, training and research needs before the hasty imposition of rigid frameworks and category “boxes.”
6. Test and re-test, until we find models that suit our communities without “throwing the baby out with the bathwater.” There is no one model that fits all.

## Conclusion

This article has been a personal reflection on neocolonialism and science diplomacy while looking at mental health policy and practice in very different Middle Eastern settings. It has been argued that true clinical, training and research excellence are those activities that meet and fulfil the clinical, training and research needs of the community in which they are embedded. There is an Arabic expression “to stretch your legs according to the length of your blanket” which is perhaps most equivalent to the English expression “to cut your coat according to your cloth.” Approaches which impose frameworks without testing and trialing for validity and utility in their new setting, risk not only failing in their mission to achieve excellence, but may, in addition, sap or re-direct vital resources away from the needs of the community. By listening carefully and taking a “bottom-up” approach, working as partners with people and organizations, more novel and innovative approaches can be adopted. Being more attuned to their setting, these approaches allow for solutions, whether in policies or clinical practice, that work in our communities for the betterment of our mental health and well-being. Ultimately, this may lead to the desired “re-balancing” of the playing field with the Global North reaching out to learn from policies and practices in the Global South. Science diplomacy could further lead to strengthening the bridges between us as we strive to tackle global dilemmas as citizens of a truly global community.

## Data availability statement

The original contributions presented in the study are included in the article/supplementary material, further inquiries can be directed to the corresponding author.

## Author contributions

ND: Writing – original draft, Writing – review & editing.

## Footnotes

^1^Principal author for both these strategy documents.

^2^United Nations concept of global citizenship is embedded in Sustainable Development Goal (SDG) 4 whereby the international community agreed to ensure learners acquire the knowledge and skills to promote sustainable development.

^3^Dajani R, Bayoumi R, Flowe H, Rockowitz S, Stevens L. (2024) ‘Neocolonialism and Science Diplomacy: Lessons Learned from the Field and the Way Forward’. Manuscript submitted for publication.

^4^Abu Dhabi was ranked as the leading city in response to Covid-19 globally, among other 50 cities in response to the COVID-19 pandemic, according to the London-based analytics consortium, Deep Knowledge Group (DKG).

^5^Dr. Sylvie Mansour formerly at the Department of Public and Community Health in Birzeit University in the West Bank lead by Professor Rita Giacaman. PhD supervision was by Professor Murray Last at University College, London.

^6^Tony Jaffe – panel discussion at International Eating Disorders conference in 2010s.

^7^This project was only possible due to the wise mentorship of Dr. Haitham ElBashir who stepped up as acting head for the transformation period, Hakam Kair who took on the role of Autism Lead and exceptional senior management support.

^8^RCPsych, UK is the professional body responsible for education and training and setting and raising standards in psychiatry. However, it has an international division reflecting that 30% of members live outside the UK.

^9^An example of material poverty but emotional wealth which can be contrasted with settings in the Global North?
